# Efficacy and safety of RC48-ADC in HER2-positive and HER2-low metastatic breast cancer: a multicenter, real-world study

**DOI:** 10.3389/fonc.2024.1435485

**Published:** 2024-11-08

**Authors:** Fei Qu, Rongrong Lu, Xinyu Wu, Qian Liu, Mengyao Zha, Huihui Li, Yuan Yuan, Zhengxiang Han, Dongyan Cai, Xiang Huang, Yongmei Yin, Wei Li

**Affiliations:** ^1^ Department of Oncology, The First Affiliated Hospital of Nanjing Medical University, Nanjing, China; ^2^ The First Clinical College of Nanjing Medical University, Nanjing, China; ^3^ Department of Breast Medical Oncology, Shandong Cancer Hospital and Institute, Shandong First Medical University and Shandong Academy of Medical Sciences, Jinan, China; ^4^ Department of Chemotherapy, Jiangsu Cancer Hospital, Jiangsu Institute of Cancer Research, The Affiliated Cancer Hospital of Nanjing Medical University, Nanjing, China; ^5^ Department of Oncology, The Affiliated Hospital of Xuzhou Medical University, Xuzhou, China; ^6^ Wuxi Cancer Institute, Affiliated Hospital of Jiangnan University, Wuxi, China; ^7^ Jiangsu Key Lab of Cancer Biomarkers, Prevention and Treatment, Collaborative Innovation Center for Personalized Cancer Medicine, Nanjing Medical University, Nanjing, China

**Keywords:** breast cancer, antibody-drug conjugates, Disitamab Vedotin, human epidermal growth factor receptor 2, efficacy, safety

## Abstract

**Background:**

A standard treatment recommendation for third-line and subsequent treatments for advanced HER2-positive breast cancer is still missing, especially for low HER2 expression. Nevertheless, there is evidence that these patients might benefits from antibody-drug conjugates (ADCs) treatment. Therefore, this study aimed to evaluate the clinical efficacy, safety, and factors affecting efficacy of Disitamab Vedotin (RC48) for treating HER2-positive and HER2-low metastatic breast cancer (MBC) in the real-world setting.

**Methods:**

A retrospective study at five clinical sites was conducted in China, enrolling MBC patients treated with RC48 from July 01, 2021 and May 31, 2023. Patient demographics, treatment patterns, and adverse events (AEs) were recorded and analyzed.

**Results:**

A total of 154 patients were included: 104 (67.53%) patients with HER2-positive and 50 (32.47%) patients with HER2-low MBC. The median progression-free survival (mPFS) was 5.06 months. The objective response rate (ORR) and disease control rate (DCR) were 36.36% and 68.83%, respectively. HER2-positive patients exhibited a mPFS of 5.93 and an ORR of 41.35%. In contrast, patients with low-HER2 had a mPFS of 4.28 months and an ORR of 26.00%. The most common AEs included neutropenia (54.55%), increased AST (53.25%), leukopenia (51.95%), and fatigue (43.51%), mostly graded mild to moderate (grade 1-2).

**Conclusions:**

This extensive study in China demonstrated that RC48 has excellent therapeutic potential for both HER2-positive and HER2-low MBC with a favorable safety profile. The study also suggests that combination therapy significantly boosts efficacy beyond monotherapy, indicating a promising avenue for future ADC development.

## 1 Introduction

Breast cancer (BC) is currently the leading cause of global cancer incidence. At initial diagnosis, 3% to 8% of patients present with metastases, and approximately 30% of early-stage cases would progress to incurable metastasis disease, with a 5-year survival rate of merely 27% (1). Human epidermal growth factor receptor 2 (ERBB2, HER2)-positive BC, which constitutes about 15-20% of all BC cases (2), and its survival rate is low due to HER2 overexpression (immunohistochemistry [IHC] score of 3+ or IHC 2+ with a positive fluorescence *in-situ* hybridization [FISH] result), resulting in high recurrence and mortality rates (3). The introduction of Trastuzumab (Herceptin), a pioneering HER2-targeted monoclonal antibody (mAb) approved by the FDA in 2011, has significantly improved survival and altered the disease course in HER2-positive (HER2+) metastatic breast cancer (MBC), as highlighted by the H0648g (4), M77001 (5), HERA studies (6). Other molecular agents targeting HER2, such as pertuzumab and pyrotinib further lengthened PFS and overall survival (OS) of these patients (7–9).

The Food and Drug Administration (FDA) and European Medicines Agency (EMA) approval in 2013 of trastuzumab emtansine (T-DM1) for HER2+ MBC, representing a significant turning point, spurring ADC research targeting HER2 (10). Despite resistance in a notable subset of patients to T-DM1, the third generation of cleavable ADCs trastuzumab deruxtecan (T-DXd or DS-8201) has shown remarkable efficacy in later-line therapies for this cohort, as evidenced by the DS8201-A-J101 and DESTINY-Breast01 trials (11, 12). T-DXd also outperformed T-DM1 in the DESTINY-Breast03 trial and has consequently been established as the standard second-line therapy for HER2+ advanced breast cancer (ABC) at present (13). It should also be noted that although T-DXd has the potential to result in a higher incidence of interstitial lung disease (10%), its safety profile is still manageable (14). Nevertheless, the absence of a standardized treatment protocol after T-DXd failure indicates that significant clinical needs remain unmet.

Disitamab Vedotin (RC48), a novel ADC from China, combines a humanized anti-HER2 monoclonal antibody (mAb) with the cytotoxic agent monomethyl auristatin E (MMAE), exhibiting potential against both high (defined as IHC 3+ or IHC 2+&FISH+) and low HER2-expressing (IHC 2+&FISH- or IHC 1+) tumors (15). Preclinical studies suggest its dual-action mechanism—disruption of microtubule formation and a bystander effect on adjacent tumor cells—regardless of their HER2 status (16, 17). Clinical insights from the C001 and C003 CANCER studies reveal promising remission rates and controllable safety in treated ABC patients (18). The mPFS was 5.5 months and 5.7 months in HER2-positive (70 cases) and HER2-low (48 cases) subgroups. Specifically, in the IHC 2+/FISH- subgroup, the ORR was 42.9% with a mPFS of 6.6 months, while even in HER2 IHC 1+ patients, the ORR and mPFS reached 30.8% and 5.5 months, respectively. The most frequently reported AEs included elevated enzyme activities, hypoesthesia, and decreased white blood cell and neutrophil counts. However, the clinical data of treatment with RC48 as third-line therapy remain sparse. Therefore, this study aimed to investigate the clinical efficacy and safety of RC48 across HER2 statuses.

## 2 Materials and methods

### 2.1 Study design and patients

This multicenter, non-interventional, retrospective study included patients with metastatic breast cancer (MBC) who were treated with at least one cycle of RC48 at five public oncology clinics across the country between June 1, 2021, and May 31, 2023, and who met predetermined enrollment criteria, with a follow-up that ended on October 31, 2023 (**Supplementary Table S1**).

Data were collated from medical records, nursing flow sheets, physician notes, orders, examination reports, and laboratory test forms. Women aged 18 years or older were considered eligible. Inclusion criteria comprised: (1) metastatic breast cancer confirmed through histopathology or images; (2) HER2-positive or low status; (3) presence of at least one measurable extracranial lesion or osteolytic or mixed bone metastases in accordance with the Response Evaluation Criteria in Solid Tumors v. 1.1 (RECIST 1.1) (19); and (4) the clinical data were complete and traceable. Exclusion criteria included previous malignancies of different histologic origins or previous treatment with RC48 in neoadjuvant or adjuvant therapy.

TNM staging adhered to the American Joint Committee on Cancer (AJCC) eighth edition (20). HER2 positivity required at least one pathological confirmation of primary or metastatic sites by participating hospitals’ pathology departments, with an IHC score of 3+ or 2+ with positive FISH (21, 22). HER2-low expression denoted HER2 IHC 1+ or 2+ without gene amplification. Estrogen (ER) and progesterone receptor (RP) statuses were determined by IHC, with a threshold of >1% tumor cells staining (23). Moreover, the disease-free interval (DFI) was defined as the time interval from radical surgery or end of curative-intent treatment to the first recurrence of the tumor (contralateral primary breast cancer, locoregional or distant recurrence). For patients with initial stage IV diagnoses, the DFI was characterized as the interval between the primary treatment of a malignancy and the first documentation of disease progression.

The research was carried out following the principles of the Declaration of Helsinki. Given its retrospective nature and adherence to legal and institutional standards, informed consent was not required. The ethics committee and institutional review board of the First Affiliated Hospital of Nanjing Medical University approved this study (No. 2023–SR-491). This study was also registered at Clinicaltrials.gov (NCT06168227).

### 2.2 Efficacy and safety evaluation

Baseline was established as the visit preceding the initiation of RC48 therapy. The assessment of tumor response was carried out based on the RECIST v1.1. Overall survival (OS), progression-free survival (PFS), disease control rate (DCR), and objective response rate (ORR) served as efficacy outcomes. PFS spanned from the first RC48 dose to the earliest data of documented progression, death from any cause, or the last follow-up. OS was the interval from the first drug administration to death. ORR represented the proportion of patients achieving complete (CR) or partial response (PR), while DCR also included stable disease (SD). Adverse events (AEs) were monitored and graded according to the National Cancer Institute’s Common Terminology Criteria for Adverse Events (CTCAE version 5.0).

### 2.3 Statistical analyses

The patients were categorized into two groups based on the expression of HER2 as the HER2-positive and HER2-low groups. Data were described as the median (range) or frequencies as appropriate. Comparison of quantitative variables between the study groups was done using t-test and Mann–Whitney U-test for parametric and nonparametric variables, respectively. For comparing categorical data, Chi-square (χ2) test was used. Survival endpoints were estimated using the Kaplan-Maier estimator and tested by the stratified log-rank test. For all analyses, a p-value less than 0.05 was deemed statistically significant at the significance level. The statistical software SPSS 25.0 was utilized for all statistical analyses.

## 3 Results

### 3.1 Basic characteristics

A total of 154 patients, with a median age of 53 (range, 28 – 84) years were enrolled. Detailed patient characteristics are presented in **Table 1**. The majority (77.27%) had an ECOG performance status of 0-1, indicating good health. Initial staging distribution varied, with 15.58% presenting with *de novo* metastatic disease and the most prevalent stages being T2 (42.86%) and N3 (25.57%). Multiple metastatic sites were common, with lymph nodes (74.68%) and bones (51.95%) being frequently involved. Brain metastases were present in 35.06% of patients. Patients had undergone a median of 3 previous chemotherapy regimens (range, 0–10), and 89.61% had received two or more lines of treatment, suggesting extensive pretreatment. HER2-positive MBC was diagnosed in 67.53%, and 32.47% exhibited low HER2 expression. Differences in baseline characteristics between HER2-positive and HER2-low groups were notable only in histological grading and PR status. HER2-positive patients predominantly had prior treatments with trastuzumab or pertuzumab (96/104), and a significant proportion (94.23%) had received prior pyrotinib or lapatinib. ER and/or PR positivity was observed in 35/50 (70.00%) patients with HER2-low MBC, with 34/50 (68.00%) of patients receiving endocrine therapy.

**Table 1:** Patient baseline demographic and disease characteristics.

Characteristic Patients	Overall population (N = 154)	HER2-positive (N = 104)	HER2-low (N = 50)	*p*-Values
Age, n (%)				0.326
Median (range)	53 (28,84)	53 (28,84)	52 (28,78)	
<60 years	124 (80.52)	86 (82.69)	38 (76.00)	
≥60 years	30 (19.48)	18 (17.31)	12 (24.00)	
ECOG status, n (%)				0.794
0–1	119 (77.27)	81 (77.88)	38 (76.00)	
≥2	35 (22.73)	23 (22.12)	12 (24.00)	
Disposition of diagnosis, n (%)				0.921
Recurrent from earlier stages, stages I–III	130 (84.42)	88 (84.62)	42 (84.00)	
*De novo*, newly diagnosed stage IV	24 (15.58)	16 (15.38)	8 (16.00)	
Histological grading, n (%)				0.020
G I	1 (0.65)	1 (0.96)	0 (0)	
G II	57 (37.01)	32 (30.77)	25 (50.00)	
G III	70 (45.45)	54 (51.92)	16 (32.00)	
unknow	26 (16.88)	17 (16.35)	9 (18.00)	
Burden of primary tumor lesion, n (%)				0.475
<5cm	99 (64.29)	68 (65.38)	31 (62.00)	
≥5cm	25 (16.23)	19 (17.59)	6 (12.00)	
unknow	30 (19.48)	17 (16.35)	13 (26.00)	
Regional lymph node involvement, n (%)				0.372
No	24 (15.58)	14 (13.46)	10 (20.00)	
Yes	118 (76.62)	80 (76.92)	38 (76.00)	
unknow	12 (7.79)	10 (9.62)	2 (4.00)	
ER status, n (%)				0.158
<1% (negative)	68 (44.16)	50 (48.08)	18 (36.00)	
≥1% (positive)	86 (55.84)	54 (51.92)	32 (64.00)	
PR status, n (%)				<0.001
<1% (negative)	84 (54.55)	66 (63.46)	18 (36.00)	
≥1% (positive)	70 (45.45)	38 (36.54)	32 (64.00)	
HER2 status, n (%)				NA
IHC 1+	17 (11.04)	0 (0)	17 (34.00)	
IHC 2+/FISH-	33 (21.43)	0 (0)	33 (66.00)	
IHC 2+/FISH+	30 (19.48)	30 (28.85)	0 (0)	
IHC 3+	74 (48.05)	74 (71.15)	0 (0)	
Ki67 index, n (%)				0.335
Low (<15%)	19 (12.34)	11 (10.58)	8 (16.00)	
High (≥15%)	129 (83.77)	89 (85.58)	40 (80.00)	
unknow	6 (3.90)	4 (3.85)	2 (4.00)	
Disease-free interval				0.445
0–12 months	59 (38.31)	42 (40.38)	17 (34.00)	
>12 months	95 (61.69)	62 (59.62)	33 (66.00)	
Number of metastasis sites, n (%)				0.219
Median (range)	4 (1,11)	4 (1,11)	4 (1,7)	
Distribution, n (%)				
<3	60 (38.96)	44 (42.31)	16 (32.00)	
≥3	94 (61.04)	60 (57.69)	34 (68.00)	
Metastatic site, n (%)				
Lymph nodes	115 (74.68)	75 (72.12)	40 (80.00)	0.292
Liver	75 (48.70)	47 (45.19)	28 (56.00)	0.209
Brain	54 (35.06)	40 (38.46)	14 (28.00)	0.203
Lung	76 (49.35)	52 (50.00)	24 (48.00)	0.816
Bone	80 (51.95)	53 (50.96)	27 (54.00)	0.724
Visceral metastases, n (%)				0.314
Yes	95 (61.69)	67 (64.42)	28 (56.00)	
No	59 (38.31)	37 (35.58)	22 (44.00)	
Lines of advanced systematic therapy of RC48, n (%)				0.456
Median no. of lines (range)	4 (1,11)	4 (1,11)	4 (1,7)	
1L	3 (1.95)	1 (0.96)	2 (4.00)	
2L	13 (8.44)	9 (8.65)	4 (8.00)	
≥3L	138 (89.61)	94 (90.38)	44 (88.00)	
Previous neoadjuvant chemotherapy, n (%)				0.295
Yes	41 (26.62)	25 (24.04)	16 (32.00)	
No	113 (73.38)	79 (75.96)	34 (68.00)	
Previous adjuvant chemotherapy, n (%)				0.793
Yes	115 (74.68)	77 (74.04)	38 (76.00)	
No	39 (25.32)	27 (25.96)	12 (24.00)	
Previous cancer treatment of advanced disease, n (%)				
Endocrine therapy	81 (52.60)	47 (45.19)	34 (68.00)	0.008
CDK4/6 inhibitor	45 (29.22)	17 (16.35)	28 (56.00)	NA
Trastuzumab or Pertuzumab	96 (62.34)	96 (92.31)	0 (0)	NA
TKIs	98 (63.64)	98 (94.23)	0 (0)	NA
Previous other ADCs therapy, n (%)				0.002
Yes	27 (17.53)	25 (24.04)	2 (4.00)	
No	127 (82.47)	79 (75.96)	48 (96.00)	

ECOG, Eastern Cooperative Oncology Group; G, grade; ER, estrogen receptor; PR, progesterone receptor; HER2, human epidermal growth factor receptor-2; IHC, immunohistochemistry. ISH, *in-situ* hybridization; CDK 4/6, cyclin-dependent kinase 4/6; TKIs, tyrosine kinase inhibitors; ADCs, antibody-drug conjugates.

Monotherapy with RC48 was chosen for 70.13% of the cohort, while the remainder received combination regimens. The combinations included RC48 with anti-angiogenic drugs 23 (50.00%), TKIs 16 (34.78%), and chemotherapy 7 (15.22%). Further treatment details are presented in **Supplementary Figure S1**.

### 3.2 Treatment effectiveness and subgroup analysis

#### 3.2.1 Overall population

**Figure 1:**
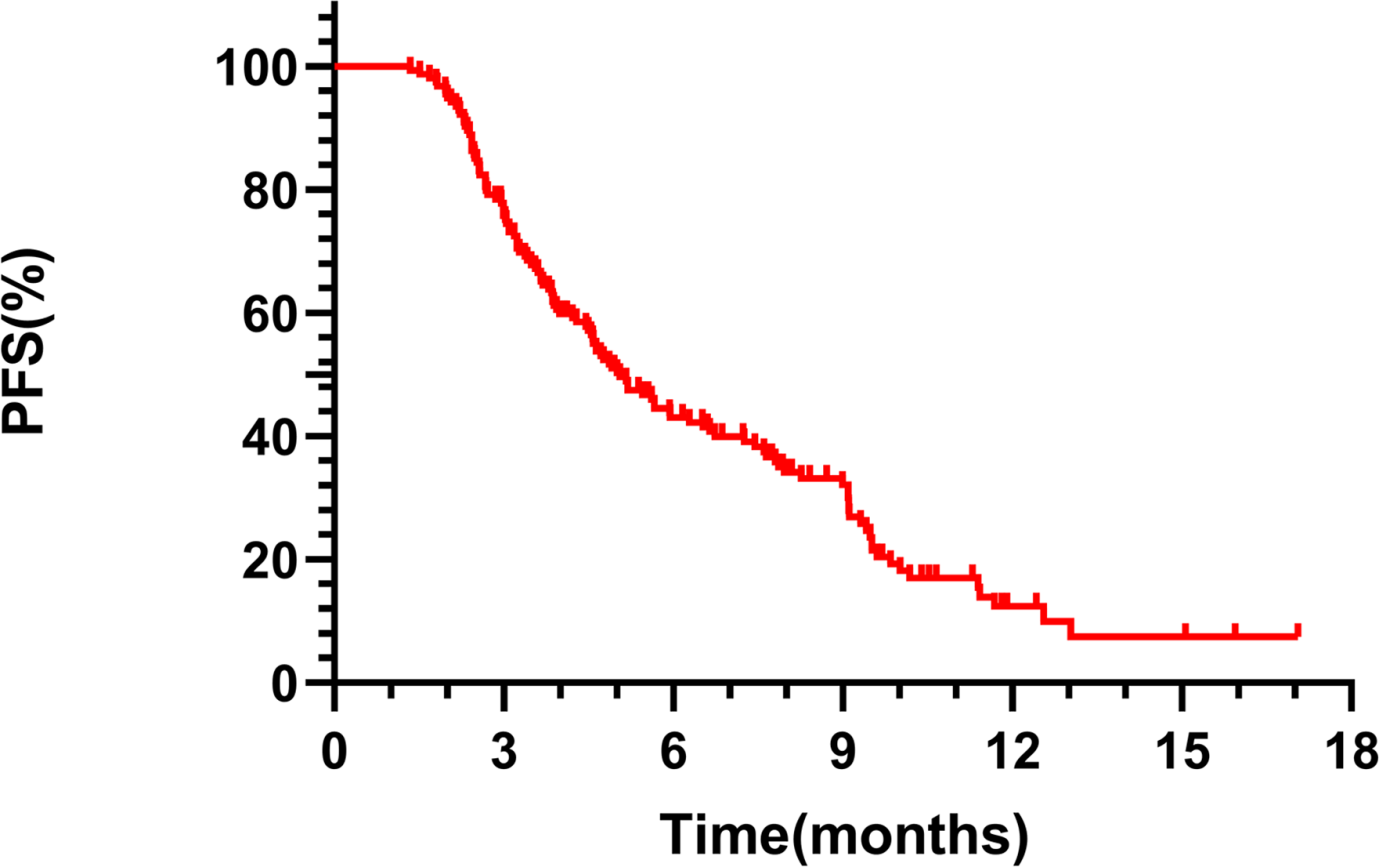
Kaplan-Meier analysis of PFS in patients treated with RC48 in the entire population.

**Figure 2:**
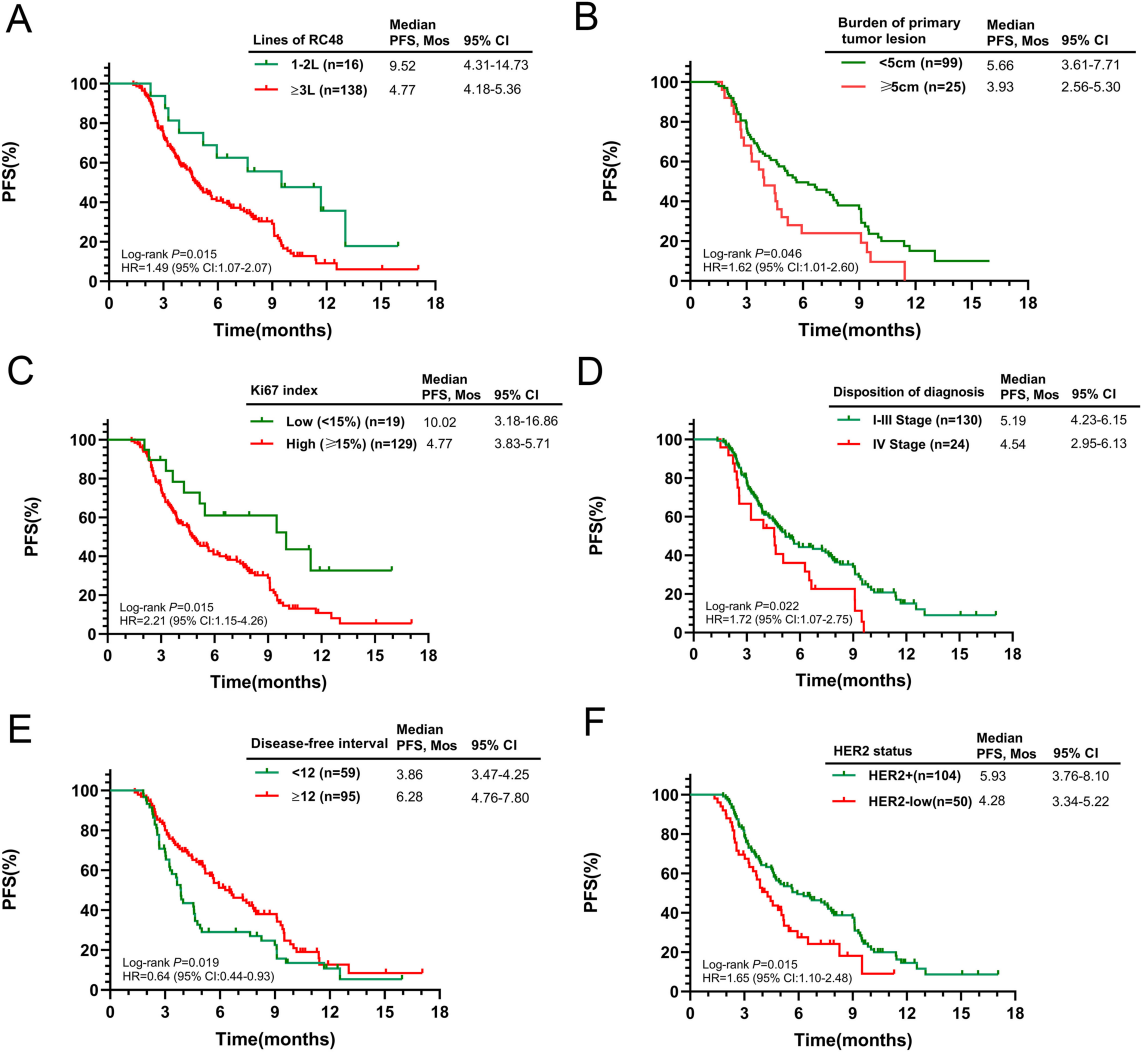
Kaplan-Meier curves for PFS according to potential predictive factors in the entire population. **(A)** Number of RC48 lines; **(B)** Burden of primary tumor lesion; **(C)** Ki67 index; **(D)** Initial diagnosis stage; **(E)** Disease-free interval; **(F)** HER2 status.

The mPFS for the overall cohort was 5.06 months (95% CI 4.24-5.88, **Figure 1**). Among the 149 patients assessed for ORR and DCR, treatment responses varied: CR was achieved in 4 patients, PR in 52, SD in 50, and PD in 43, resulting in an ORR of 36.36% and a DCR of 68.83%. Notably, patients receiving RC48 as first- or second-line therapy had a longer mPFS (9.52 months) compared to those treated in third or subsequent lines (4.77 months, p = 0.0015, **Figure 2A**). In the first- and second-line settings, ORR and DCR were 43.75% and 75.00%, respectively. In contrast, for patients treated in third-line or beyond, the ORR was 35.50% and DCR was 68.12%.

Subsequent analysis explored the relationship between baseline characteristics and PFS. Patients with smaller primary tumors (≤5cm) and a lower Ki-67 index (≤14%) had significantly longer survival (p = 0.046 and p = 0.015, respectively). Conversely, those with Stage IV cancer at diagnosis experienced a shorter mPFS when treated with RC48 (p = 0.022). Recurrence or progression within a year post-diagnosis was associated with a reduced mPFS (3.86 months) compared to those progressing after one year (6.28 months, p = 0.019). HER2-positive patients had a mPFS of 5.93 months (95% CI 3.76–8.10), while those with low HER2 expression had 4.28 months (95% CI 3.34–5.22, p = 0.015). Detailed survival outcomes across subgroups are presented in **Figures 2B–F**. No significant differences in median PFS were observed when stratified by age, ECOG score, BMI, regional lymph node involvement, ER status, PR status, number of metastatic sites, or site-specific metastases (**Supplementary Figure S2**).

#### 3.2.2 HER2-positive MBC participants

In the HER2-positive cohort, the mPFS was 5.93 months (95% CI 3.76–8.10), with an ORR of 41.35% and a DCR of 71.15%. Follow-up results showed no significant survival difference between patients with HER2 IHC 3+ and IHC 2+/FISH+ statuses, with mPFS of 5.61 and 6.28 months, respectively (p = 0.914). Higher ECOG scores and Ki-67 indices were associated with reduced RC48 efficacy (p = 0.032 and p = 0.019). Those who received first-line chemotherapy alone after recurrence showed a significantly longer mPFS (9.52 months) than those treated with second-line or higher chemotherapy (4.77 months, p = 0.016). Patients with no previous TKI treatments saw a non-significant increase in mPFS to 11.68 months (p = 0.106) (**Supplementary Table S2**).

#### 3.2.3 HER2-low MBC participants

For participants with HER2-low MBC, mPFS was 4.28 months (95% CI 3.34–5.22), with an ORR of 26.00% and DCR of 64.00%. Subgroup analyses yielded similar findings (**Supplementary Table S3**). Presence of regional lymph node metastasis was associated with a reduced mPFS of 3.86 months, versus 8.26 months for patients without such metastasis (p = 0.045). The mPFS also varied with hormone receptor (HR) status; ER-positive patients had a mPFS of 5.16 months compared to 2.56 months for ER-negative patients (p < 0.001), and PR-positive patients had a mPFS of 5.06 months versus 3.23 months for PR-negative patients (p = 0.004). That surprised the population that those with liver metastasis displayed improved prognosis (5.45 months vs. 3.02 months, p = 0.006).

### 3.3 Treatment patterns

**Figure 3:**
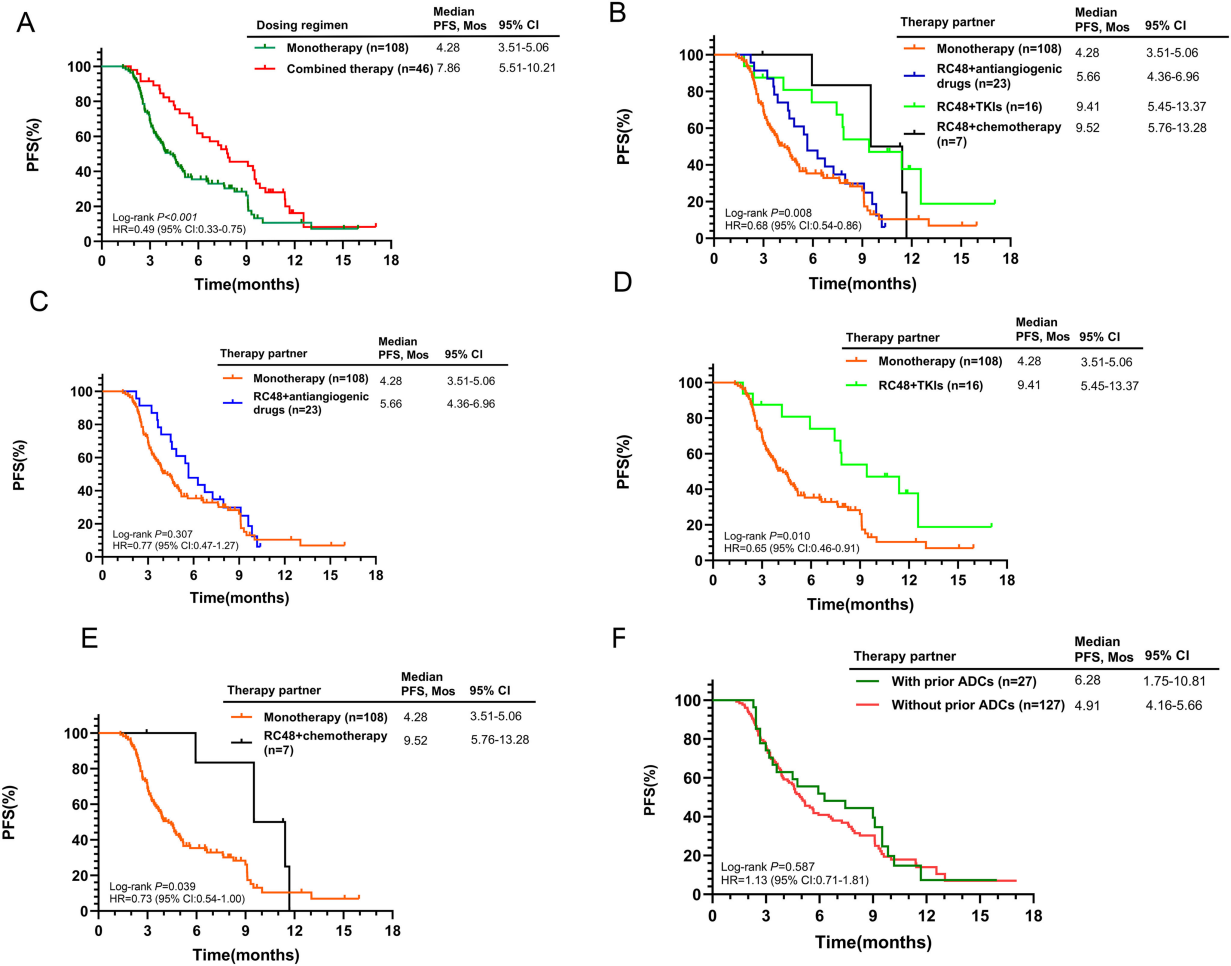
Kaplan-Meier curves for PFS according to different treatment characteristics in the entire population. **(A)** RC48 monotherapy vs. Combined therapy; **(B)** Comparison of monotherapy and different combination regimens; **(C)** RC48 monotherapy vs. RC48+antiangiogenic drugs; **(D)** RC48 monotherapy vs. RC48+TKIs; **(E)** RC48 monotherapy vs. RC48+chemotherapy; **(F)** With prior ADCs vs. Without prior ADCs.

Initial findings from the 154-patient cohort indicated superior efficacy in the combined treatment group versus RC48 alone (7.86 months vs. 4.28 months, p<0.001, **Figure 3A**). The mPFS for combination therapies was 5.66 months with antiangiogenic drugs, 9.41 months with TKIs, and 9.52 months with chemotherapy (**Figure 3B**). The RC48 combined with TKIs or chemotherapy groups showed better outcomes compared to RC48 monotherapy (p = 0.010 and p = 0.039, respectively, **Figures 3C–E**). Prior use of other ADCs did not show a significant difference in mPFS (6.28 months with prior ADCs vs. 4.91 months without, p = 0.587, **Figure 3F**). Subgroup analysis revealed that HER2-positive patients who received RC48 as first- or second-line therapy and in combination regimens experienced significantly better therapeutic efficacy (**Supplementary Table S4**).

### 3.4 Exploratory analyses

Of patients who discontinued study treatment, 53 (65.4%) of 81 in the HER2-positive cohort and 30 (83.3%) of 36 in the HER2-low cohort could receive subsequent treatment information (**Supplementary Table S5**). The systemic cancer treatment in the HER2+ subgroup included anti-HER2 monoclonal antibodies (15 [28.3%]), pyrotinib (15 [28.3%] of 53), T-DM1 (9 [17.0%]) and DS-8201 (13 [24.5%]). In the treatment of HER2-low subgroup, 12 (40.0%) of 30 patients received other single-agent chemotherapy, eight (26.7%) received taxane/platinum combination regimens, four (13.3%) received sacituzumab govitecan, three (10.0%) received DS-8201 and two (6.7%) received SKB264 after dis-continuing study treatment.

### 3.5 Safety

Patient adverse events are detailed in **Table 2**. A significant majority (96.10%) experienced at least one adverse event. The most frequent adverse events across all grades were neutropenia (54.55%), elevated levels of aspartate aminotransferase (AST) (53.25%), leukopenia (51.95%), anemia (46.10%), and asthenia (43.51%). Of note, 24.03% of patients encountered serious adverse events (grade 3/4), primarily marked by a substantial decrease in neutrophil count (16.23%) and white blood cell (WBC) count (11.69%). Mild to moderate electrolyte imbalances were also observed, with hyponatremia affecting 14.29% of patients (N = 22) and hypokalemia affecting 7.14%. The overall incidence of AEs in the monotherapy and combined treatment groups was observed, focusing on major AEs affecting at least 30% of the total population. The incidence of neutropenia, leukopenia, elevated AST levels and constipation in the combined treatment group was slightly higher than that in the monotherapy group.

**Table 2:** The summary of treatment-related adverse events.

Events	All (N = 154)		Monotherapy (N = 108)		Combined therapy (N = 46)	
	All Grades, n (%)	Grade 3/4, n (%)	All Grades, n (%)	Grade 3/4, n (%)	All Grades, n (%)	Grade 3/4, n (%)
Neutrophil count decreased	84 (54.55)	25 (16.23)	53 (49.07)	15 (13.89)	31 (67.40)	10 (21.74)
AST increased	82 (53.25)	1 (0.65)	64 (59.26)	1 (0.93)	18 (39.13)	–
WBC count decreased	80 (51.95)	18 (16.9)	50 (46.30)	11 (10.19)	30 (65.22)	7 (15.22)
Lymphocyte count decreased	71 (46.10)	2 (1.30)	45 (41.67)	1 (0.93)	26 (56.52)	1 (2.17)
Anemia	71 (46.10)	3 (1.95)	48 (44.44)	2 (1.85)	23 (50.00)	1 (2.17)
Asthenia	67 (43.51)	1 (0.65)	43 (39.81)	1 (0.93)	24 (52.17)	–
Appetite loss	60 (38.96)	–	37 (34.26)	–	23 (50.00)	–
Constipation	57 (37.01)	2 (1.30)	34 (31.48)	2 (1.85)	23 (50.00)	–
ALT increased	50 (32.47)	3 (1.95)	41 (37.96)	2 (1.85)	9 (19.57)	1 (2.17)
Blood LDH increased	40 (25.97)	–	31 (28.70)	–	9 (19.57)	–
Abdominal distention and diarrhea	40 (25.97)	2 (1.30)	21 (19.44)	1 (0.93)	19 (41.30)	1 (2.17)
Platelet count decreased	34 (22.08)	1 (0.65)	24 (22.22)	–	10 (21.74)	1 (2.17)
Dyslipidemia	33 (21.43)	–	26 (24.07)	–	7 (15.22)	–
Limb soreness	33 (21.43)	–	25 (23.15)	–	8 (17.39)	–
Blood ALP increased	30 (19.48)	1 (0.65)	24 (22.22)	–	6 (13.04)	1 (2.17)
Hyperuricuria	29 (18.83)	–	18 (16.67)	–	11 (23.91)	–
Weight loss	28 (18.18)	–	17 (15.74)	–	11 (23.91)	–
Ocular adverse effects	27 (17.53)	–	17 (15.74)	–	10 (21.74)	–
Lnsomnia	25 (16.23)	–	15 (13.89)	–	10 (21.74)	–
Nausea and vomiting	23 (14.94)	–	15 (13.89)	–	8 (17.39)	–
Blood GGT increased	22 (14.29)	–	15 (13.89)	–	7 (15.22)	–
Hyponatremia	22 (14.29)	1 (0.65)	13 (12.04)	–	9 (19.57)	1 (2.17)
Hypoalbuminemia	21 (13.64)	–	13 (12.04)	–	8 (17.39)	–
Swelling and aching of gingiva	21 (13.64)	–	13 (12.04)	–	8 (17.39)	–
Hair loss	16 (10.39)	–	10 (12.04)	–	6 (13.04)	–

This table shows adverse events occurring in at least 10% of patients from the initiation to day 28 after the last treatment. AST, aspartate aminotransferase; WBC, white blood cell; ALT, alanine aminotransferase; LDH, lactate dehydrogenase; ALP, alkaline phosphatase; GGT, γ -glutamyltransferase.

## 4 Discussion

RC48 has demonstrated exceptional efficacy in multiple malignancies with varying HER2 expression. In this multicenter, retrospective study, we assessed the efficacy and safety of RC48 in Chinese patients with HER2-positive or HER2-low MBC. Data collected up until October 2023 showed a mPFS of 5.06 months across the cohort. ORR was 36.36%, and DCR was 68.83%. Our findings suggest that patients with smaller tumors, lower Ki67 indices, and treatment at earlier lines had improved PFS. Notably, initial stage of diagnosis influenced PFS, possibly due to variations in treatment across recurrent and *de novo* metastatic breast cancer cases. Regarding the RC48 safety profile, hematological abnormalities were the most frequently observed AEs, with rates of decreased WBC count, neutrophil count, and anemia all exceeding 40% in this study. Liver and gastrointestinal issues were also common but mostly mild and manageable.

Patients were stratified by their documented HER2 status. Among those with HER2+ tumors, the mPFS was 5.93 months and ORR was 41.35%. Importantly, no significant mPFS disparities were found between HER2 IHC 2+/FISH+ and IHC 3+ patients. The mPFS was comparable to that observed with T-DM1, neratinib, and lapatinib. Moreover, RC48 demonstrated a favorable safety profile, characterized by a lower incidence of AEs relative to other ADCs, including T-DXd and U3-1402. Most HER2-positive patients had previously received TKIs and subgroup analysis indicated no significant mPFS difference between patients with or without prior TKI treatment (5.66 months vs. 11.68 months, P = 0.106). These observations may be due to the different anti-tumor mechanisms of ADCs and TKIs. While monoclonal antibodies and TKIs impede HER2-positive tumor proliferation by blocking the HER2 signaling pathway, mutations in PIK3CA, absence of PTEN, and alternative signaling pathways may reduce TKI efficacy (24, 25). Conversely, RC48’s dual-action mechanism (26)—antibody activity and cytotoxic payload release—differs from TKI resistance pathways (27, 28). However, concerns have been raised regarding the failure of paclitaxel in breast cancer clinical trials, attributed to paclitaxel resistance mediated by p-glycoprotein, which reduces intracellular drug concentration. Researchers found that MMAE is much more toxic than paclitaxel at the same concentrations, potentially counteracting the transport of p-glycoprotein and preserving its therapeutic efficacy. On the other hand, Disitamab, a HER2 antibody from RC48, activates the cGAS-STING pathway, boosting IFN-β secretion. This enhances immune cell infiltration and strengthens anti-tumor immunity (29).

Increasing recognition has been given to the prevalence of tumors exhibiting low or heterogeneous HER2 expression. HER2-low breast cancer, defined as IHC 1+ or IHC 2+ and FISH-, accounts for approximately 45%-55% of all breast tumors, with a higher proportion in HR+ patients (30). In China, considering factors such as accessibility and affordability, cytotoxic chemotherapy remains mainstay of treatment for patients with HER2-low MBC who have failed prior therapies, including endocrine therapy (31). For patients previously treated with anthracyclines and taxanes, monochemotherapy was correlated with only 2.8-4.2 months of PFS (32). For second-line therapy, the NCCN guidelines now recommend T-DXd, an innovative HER2-targeted ADC, as the preferred treatment for patients with HER2-low MBC, based upon the DESTINY Breast-04 trial, presented in 2022 (33). In this study, RC48 had a mPFS of 4.28 months and an ORR of 26.00% in HER2-low patients, aligning with phase I/II trial (NCT02277717/NCT04742153) outcomes of SYD985 (34) and MRG002 (35). This result was lower than that of HER2-positive patients, which may be attributed to the reduced HER2 expression in tumour cells and small sample size of this subgroup. Taken together, RC48 has shown promising efficacy and safety in the treatment of HER2+ and HER2-low MBC, especially in patients with liver metastases.

ADC monotherapy’s efficacy is limited by resistance mechanisms, hence ongoing research aims to combine ADCs with other anticancer drugs to extend clinical benefits. In our study, combination treatments resulted in longer mPFS than monotherapy (7.86 months vs. 4.28 months), suggesting that optimal therapeutic partnerships could enhance antitumor activity. In preclinical models, combining gemcitabine with ADCs may increase HER2 expression and, consequently, ADC effectiveness (36). Furthermore, antiangiogenics may improve ADC penetration and tumor cell exposure (37–40). Furthermore, because the two combination treatments, namely RC48+TKIs (mPFS: 9.41 months) and RC48+chemotherapy (mPFS: 9.52 months), had similar benefits and there were fewer patients who got RC48+chemotherapy than those who got TKIs combined with RC48 in our trial, ADC with TKI might work better than ADC alone or other combination treatments, meaning that specific TKIs could be more compatible with ADCs. The addition of a TKI to achieve dual target blockade could offer greater specificity and potentially improve the therapeutic index (41); however, this result may not be generalizable to other ADC-TKI pairings or to settings where HER2 is not involved. At the same time, besides assessing the efficacy of drugs, we also need to consider that a combination of multiple drugs means a higher chance of side effects and toxicity. Thus, a strict head-to-head randomized controlled trial (RCT) is needed to confirm the benefits of better combination therapies.

Our study acknowledges several limitations arising from its design and methodology, including a modest sample size, the retrospective nature of the analysis and the possibility of selection bias due to physicians’ preferences in treatment choices. Moreover, factors such as scheduling of follow-up visits, patient compliance with treatment regimens, and inconsistencies in evaluating treatment responses could have influenced the PFS outcomes. The scope of this study did not extend to a comparative analysis of RC48’s effectiveness and safety relative to existing third-line treatments for advanced breast cancer.

Despite these constraints, our study’s strengths lie in its multicenter approach and the real-world context of the patient cohort, enhancing the relevance and applicability of the data. We have also offered detailed accounts of subsequent treatment pathways. Furthermore, this study represents the pioneering effort to assess the clinical effectiveness of RC48 when used in conjunction with other antitumor medications. We would continue to follow up, further gather extensive long-term survival data and investigate the therapeutic profile of RC48.

To conclude, this extensive study in China demonstrated that RC48 has excellent therapeutic potential for both HER2-positive and HER2-low MBC with a favorable safety profile. The study also suggests that combination therapy significantly boosts efficacy beyond monotherapy. These findings in this study should be confirmed in larger, more diverse patient populations in future.

## Data Availability

The original contributions presented in the study are included in the article/**Supplementary Material**. Further inquiries can be directed to the corresponding authors.
