# Supplementary material for: Efficacy and safety of RC48-ADC in HER2-positive and HER2-low metastatic breast cancer: a multicenter, real-world study
Source: Front Oncol. 2024 Nov 8;14:1435485. doi: 10.3389/fonc.2024.1435485 (PMC11582051; doi:10.3389/fonc.2024.1435485)
Supplement: Supplementary file 1 [file DataSheet1.docx]

Supplementary Material

## Supplementary Figures


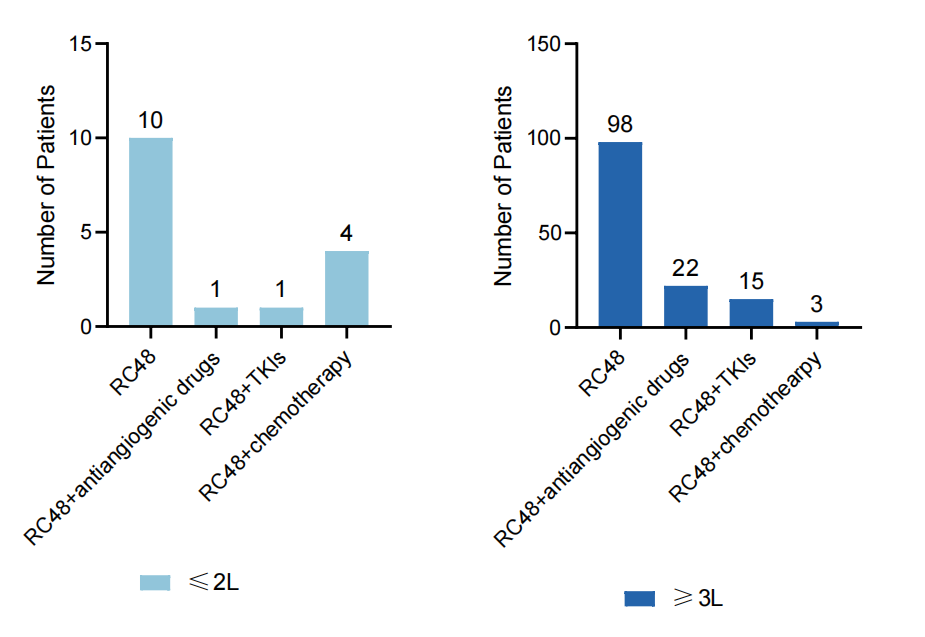


**Figure S1.** Treatment patterns in Chinese real-world population. TKIs, tyrosine kinase inhibitors.


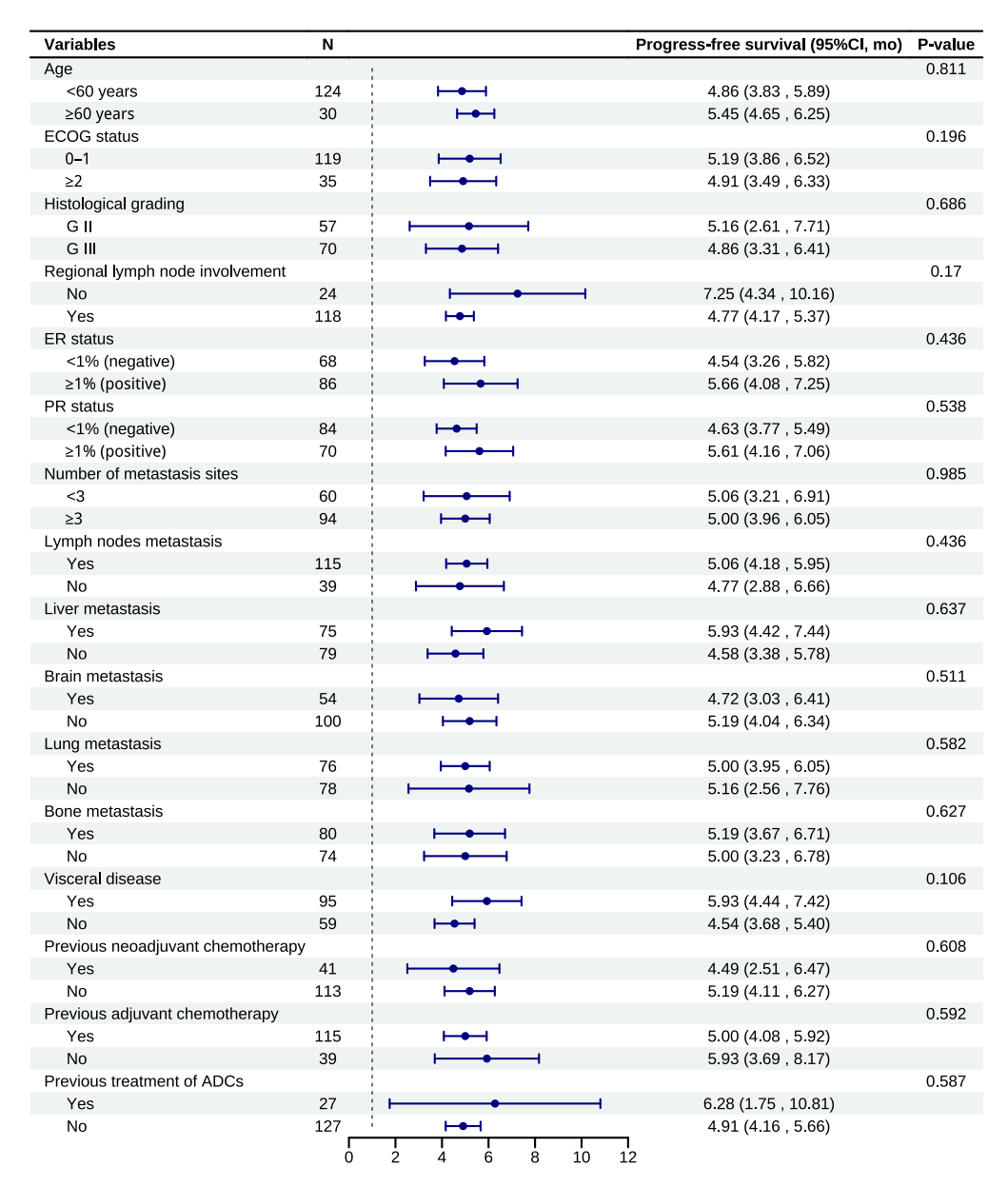


**Figure S2.** Subgroup analysis of 154 Chinese patients with HER2-positive or HER2-low metastatic breast cancer treated with RC48 according to their baseline characteristics.PFS and 95% CIs in key subgroups based on baseline characteristics. ECOG, Eastern Cooperative Oncology Group; ER, estrogen receptor; PR, progesterone receptor; CI, confidence interval; ADC, antibody-drug conjugate.

## Supplementary Tables

**Table S1.** Name of participating hospitals

| No. | Name |
| --- | --- |
| 1 | The First Affiliated Hospital with Nanjing Medical University |
| 2 | Shandong Cancer Hospital |
| 3 | Jiangsu Cancer Hospital |
| 4 | The Affiliated Hospital of Xuzhou Medical University |
| 5 | The Affiliated Hospital of Jiangnan University |

**Table S2.** Prespecified subgroup analysis of progression-free survival of the 104 Chinese patients with HER2-positive metastatic breast cancer treated with RC48.

|  | **HER2-positive (N=104)** | | |
| --- | --- | --- | --- |
| **Characteristics** | **Total (cases)** | **mPFS (95% CI) (mo)** | ***p*-Values** |
|  | 104 | 5.93 (3.76-8.10) |  |
| Age |  |  | 0.465 |
| <60 years | 86 | 5.93 (3.28-8.58) |  |
| ≥60 years | 18 | 5.66 (3.45-7.87) |  |
| ECOG status |  |  | 0.032 |
| 0-1 | 81 | 7.25 (5.35-9.16) |  |
| ≥2 | 23 | 4.58 (3.05-6.11) |  |
| Histological grading |  |  | 0.869 |
| G II | 32 | 5.61 (1.40-9.82) |  |
| G III | 54 | 5.93 (2.92-8.94) |  |
| Burden of primary tumor lesion |  |  | 0.087 |
| <5cm | 68 | 7.6 (6.30-8.90) |  |
| ≥5cm | 19 | 4.49 (3.55-5.43) |  |
| Regional lymph node involvement |  |  | 0.572 |
| No | 14 | 6.73 (3.82-9.65) |  |
| Yes | 80 | 5.93 (3.35-8.51) |  |
| Disposition of diagnosis |  |  | 0.055 |
| Recurrent from earlier stages, stages I–III | 88 | 6.73 (4.40-9.06) |  |
| De novo, newly diagnosed stage IV | 16 | 4.54 (3.17-5.91) |  |
| ER status |  |  | 0.770 |
| <1% (negative) | 50 | 5.66 (2.15-9.17) |  |
| ≥1% (positive) | 54 | 5.93 (3.65-8.21) |  |
| PR status |  |  | 0.909 |
| <1% (negative) | 66 | 5.19 (2.50-7.88) |  |
| ≥1% (positive) | 38 | 6.64 (4.38-8.90) |  |
| HER2 status |  |  | 0.914 |
| IHC 2+/FISH+ | 30 | 6.28 (4.89-7.67) |  |
| IHC 3+ | 74 | 5.61 (2.72-8.50) |  |
| Ki-67 index |  |  | 0.019 |
| Low (<15%) | 11 | 11.39 (8.95-13.84) |  |
| High (≥15%) | 89 | 5.66 (3.80-7.52) |  |
| Disease-free interval |  |  | 0.181 |
| 0–12 months | 42 | 4.58 (3.60-5.56) |  |
| >12 months | 62 | 7.44 (5.82-9.06) |  |
| Number of metastasis sites |  |  | 0.539 |
| <3 | 44 | 6.73 (3.94-9.52) |  |
| ≥3 | 60 | 5 (2.57-7.43) |  |
| Visceral disease |  |  | 0.100 |
| Yes | 67 | 7.57 (5.32-9.18) |  |
| No | 37 | 6.21 (3.41-5.85) |  |
| Lymph node metastasis |  |  | 0.628 |
| Yes | 75 | 6.28 (4.05-8.51) |  |
| No | 29 | 4.77 (2.92-6.62) |  |
| Liver metastasis |  |  | 0.585 |
| Yes | 47 | 5.93 (3.45-8.41) |  |
| No | 57 | 7.44 (4.33-10.55) |  |
| Brain metastasis |  |  | 0.443 |
| Yes | 40 | 4.91 (0.67-9.15) |  |
| No | 64 | 6.28 (4.13-8.43) |  |
| Lung metastasis |  |  | 0.594 |
| Yes | 52 | 5.61 (3.83-7.39) |  |
| No | 52 | 7.25 (3.94-10.56) |  |
| Bone metastasis |  |  | 0.671 |
| Yes | 53 | 6.28 (3.65-8.91) |  |
| No | 51 | 5.93 (3.00-8.86) |  |
| Lines of advanced systematic therapy of RC48 |  |  | 0.016 |
| ≤2L | 10 | 13.03 (7.99-18.07) |  |
| ≥3L | 94 | 5.61 (4.09-7.13) |  |
| Previous treatment of trastuzumab or pertuzumab |  |  | 0.083 |
| Yes | 96 | 5.66 (3.98-7.34) |  |
| No | 8 | 11.68 (6.87-16.49) |  |
| Previous treatment of TKIs |  |  | 0.106 |
| Yes | 98 | 5.66 (3.96-7.36) |  |
| No | 6 | 11.68 (3.25-20.11) |  |

ECOG, Eastern Cooperative Oncology Group; G, grade; ER, estrogen receptor; PR, progesterone receptor; HER2, human epidermal growth factor receptor-2; IHC, immunohistochemistry. FISH, in-situ hybridisation; TKIs, tyrosine kinase inhibitors; 95% CI, 95% confident index; mPFS, median progression-free survival; mo, months.

**Table S3.** Prespecified subgroup analysis of progression-free survival of the 50 Chinese patients with HER2-low metastatic breast cancer treated with RC48.

|  | **HER2-low (N=50)** | | |
| --- | --- | --- | --- |
| **Characteristics** | **Total (cases)** | **mPFS (95% CI) (mo)** | ***p*-Values** |
|  | 50 | 4.28 (3.34-5.22) |  |
| Age |  |  | 0.431 |
| <60 years | 38 | 3.98 (3.09-4.88) |  |
| ≥60 years | 12 | 5.06 (3.29-6.83) |  |
| ECOG status |  |  | 0.426 |
| 0-1 | 38 | 3.98 (3.05-4.91) |  |
| ≥2 | 12 | 5.45 (2.42-8.48) |  |
| Histological grading |  |  | 0.037 |
| G II | 25 | 5.06 (4.07-6.05) |  |
| G III | 16 | 3.02 (2.24-3.8) |  |
| Burden of primary tumor lesion |  |  | 0.138 |
| <5cm | 31 | 3.86 (3.07-4.65) |  |
| ≥5cm | 6 | 2.43 (1.28-3.58) |  |
| Regional lymph node involvement |  |  | 0.045 |
| No | 10 | 8.26 (4.81-11.71) |  |
| Yes | 38 | 3.86 (3.05-4.67) |  |
| Disposition of diagnosis |  |  | 0.199 |
| Recurrent from earlier stages, stages I–III | 42 | 4.28 (3.12-5.45) |  |
| De novo, newly diagnosed stage IV | 8 | 2.56 (0-5.25) |  |
| ER status |  |  | <0.001 |
| <1% (negative) | 18 | 2.56 (1.5-3.62) |  |
| ≥1% (positive) | 32 | 5.16 (3.59-6.73) |  |
| PR status |  |  | 0.004 |
| <1% (negative) | 18 | 3.23 (2.47-3.99) |  |
| ≥1% (positive) | 32 | 5.06 (4.1-6.02) |  |
| HER2 status |  |  | 0.443 |
| IHC 1+ | 17 | 4.28 (3.2-5.36) |  |
| IHC 2+/FISH- | 33 | 4.86 (2.55-7.17) |  |
| Ki-67 index |  |  | 0.212 |
| Low (<15%) | 8 | 5.16 (3.77-6.55) |  |
| High (≥15%) | 40 | 3.86 (3.27-4.45) |  |
| Disease-free interval |  |  | 0.002 |
| 0–12 months | 17 | 3.02 (1.98-4.06) |  |
| >12 months | 33 | 5.16 (4.49-5.83) |  |
| Number of metastasis sites |  |  | 0.074 |
| <3 | 16 | 2.56 (1.24-3.88) |  |
| ≥3 | 34 | 4.86 (3.73-5.99) |  |
| Visceral disease |  |  | 0.958 |
| Yes | 28 | 3.86 (2.43-5.29) |  |
| No | 22 | 4.28 (2.91-5.65) |  |
| Lymph node metastasis |  |  | 0.519 |
| Yes | 40 | 3.98 (2.99-4.97) |  |
| No | 10 | 4.28 (1.42-7.14) |  |
| Liver metastasis |  |  | 0.006 |
| Yes | 28 | 5.45 (4.1-6.8) |  |
| No | 22 | 3.02 (2.04-4) |  |
| Brain metastasis |  |  | 0.715 |
| Yes | 14 | 3.86 (2.09-5.63) |  |
| No | 36 | 4.45 (3.21-5.69) |  |
| Lung metastasis |  |  | 0.878 |
| Yes | 24 | 4.45 (3.13-5.77) |  |
| No | 26 | 3.86 (3.09-4.63) |  |
| Bone metastasis |  |  | 0.053 |
| Yes | 27 | 4.86 (3.82-5.9) |  |
| No | 23 | 3.27 (1.9-4.64) |  |
| Lines of advanced systematic therapy of RC48 |  |  | 0.243 |
| ≤2L | 6 | 5.19 (2.69-7.69) |  |
| ≥3L | 44 | 3.98 (3.03-4.94) |  |

ECOG, Eastern Cooperative Oncology Group; G, grade; ER, estrogen receptor; PR, progesterone receptor; HER2, human epidermal growth factor receptor-2; IHC, immunohistochemistry. FISH, in-situ hybridisation; 95% CI, 95% confident index; mPFS, median progression-free survival; mo, months.

**Table S4.** Subgroup analysis of therapeutic efficacy

| **HER2 status** | **Number lines of RC48** | **Treatment Patterns** | **Total (cases)** | **Effectiveness, n, %** | | | | | | | |
| --- | --- | --- | --- | --- | --- | --- | --- | --- | --- | --- | --- |
|  |  |  | 154 | CR | | PR | | SD | | PD | |
| HER2-positive | 1-2L | Monotherapy | 8 | 2 | 25% | 1 | 12.5% | 2 | 25% | 2 | 25% |
|  |  | Combined therapy | 2 | 1 | 50% | 1 | 50% | 0 | 0 | 0 | 0 |
|  | ≥3L | Monotherapy | 62 | 0 | 0 | 17 | 27.4% | 21 | 33.9% | 22 | 35.5% |
|  |  | Combination therapy | 32 | 1 | 3.1% | 20 | 62.5% | 8 | 25% | 3 | 9.4% |
| HER2-low | 1-2L | Monotherapy | 2 | 0 | 0 | 0 | 0 | 2 | 100% | 0 | 0 |
|  |  | Combination therapy | 4 | 0 | 0 | 0 | 0 | 1 | 25.0% | 1 | 25% |
|  | ≥3L | Monotherapy | 36 | 0 | 0 | 10 | 27.8% | 12 | 33.3% | 12 | 33.3% |
|  |  | Combination therapy | 8 | 0 | 0 | 1 | 12.5% | 4 | 50% | 3 | 37.5% |

RC48, disitamab vedotin; CR, complete response; PR, partial response; SD, stable disease; PD, progressive disease; HER2, human epidermal growth factor receptor 2.

**Table S5.** Post-trial cancer therapies of patients discontinued study treatment.

|  | **HER2-positive (N=53)**  **n, %** | **HER2-low (N=30)**  **n, %** |
| --- | --- | --- |
| Pyrotinib | 15 (28.3) | - |
| Pyrotinib+chemotherapy/endocrine therapy | 8 (15.1) | - |
| Pyrotinib+trastuzumab+chemotherapy | 5 (9.4) | - |
| Pyrotinib+bevacizumab+chemotherapy | 2 (3.8) | - |
| Trastuzumab+chemotherapy | 2 (3.8) | - |
| Trastuzumab+pertuzumab+chemotherapy | 11 (20.8) | - |
| Inetetamab+vinorelbine | 2 (3.8) | - |
| Endocrine therapy | 1 (1.9) | - |
| Anti-HER2 ADCs | 22 (41.5) | 3 (10.0) |
| Trastuzumab emtansine | 9 (17.0) | - |
| Trastuzumab deruxtecan | 13 (24.5) | 3 (10.0) |
| Anti-Trop2 ADCs | - | 6 (20.0) |
| Sacituzumab govitecan | - | 4 (13.3) |
| SKB264 | - | 2 (6.7) |
| Taxane/platinum combination regimens | - | 8 (26.7) |
| Other single-agent chemotherapy | - | 12 (40.0) |

HER2, human epidermal growth factor receptor 2; ADC, antibody-drug conjugate.
